# Tilapia Bone-Derived Hydroxyapatite Particles for Controlled Citronella (*Cymbopogon nardus*) Release and Antimicrobial Activity

**DOI:** 10.1021/acsomega.5c11133

**Published:** 2026-02-12

**Authors:** Janaina Tasca Serafim, Henrique Borba Modolon, Natália Morelli Possolli, Oscar R. K. Montedo, Elídio Angioletto, Maria Alice Prado Cechinel, Sabrina Arcaro

**Affiliations:** † Technical Ceramics Laboratory (CerTec), Biomaterials and Nanostructured Materials Group, Graduate Program in Materials Science and Engineering (PPGCEM), University of the Extreme South of Santa Catarina, 88806-000 Criciúma, SC, Brazil; ‡ Laboratory of Biomaterials and Antimicrobial Materials Development (LADEBIMA), University of the Extreme South of Santa Catarina, 88806-000 Criciúma, SC, Brazil; § Department of Chemical and Food Engineering (EQA), Federal University of Santa Catarina (UFSC), 88040-900 Florianópolis, SC, Brazil

## Abstract

This study investigated the use of nanostructured hydroxyapatite (HA) derived from tilapia (*Oreochromis niloticus*) bones to produce particles for encapsulating citronella essential oil within a sodium alginate matrix, aimed at antimicrobial applications. Particles were obtained by emulsifying sodium alginate, citronella essential oil, and HA in different proportions, followed by dripping the emulsion into a CaCl_2_ solution. Rheological properties were characterized by rotational rheometry to assess emulsion stability, thixotropy, and viscosity. All emulsions exhibited pseudoplastic and thixotropic behavior, with viscosity decreasing as HA concentration increased. While higher HA content improved emulsion stability, it reduced thixotropy, resulting in lower encapsulation efficiency. Release kinetics indicated that the formulation with intermediate HA content enabled more controlled essential oil release. In antimicrobial assays, this formulation achieved the highest activity against *Escherichia coli*, with an inhibition halo of 34 ± 3 mm. The results propose a sustainable strategy for enhancing controlled release and antimicrobial performance of encapsulated agents, employing biocompatible materials derived from animal waste, with potential applications in biomedicine, food preservation, and environmental protection.

## Graphical abstract

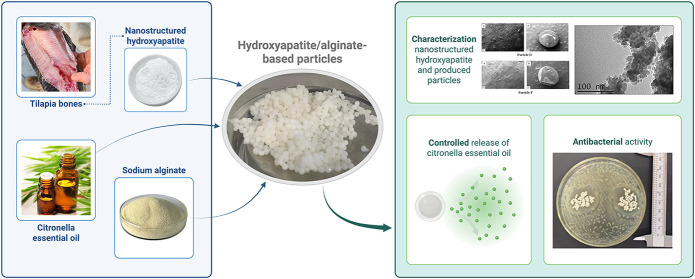

## 1 Introduction

Encapsulation is a well-established technique that provides an effective strategy for modulating the release of active ingredients such as drugs, pesticides, dyes and flavors.^1–3^ This is possible by creating a physical barrier between the core containing the active ingredient and the other components of the product. This approach aims to prevent unwanted chemical and physical reactions while preserving the biological, functional and physicochemical properties of the encapsulated material to maximize its efficacy in the application environment.^4–6^ The use of encapsulation techniques preserves the properties and promotes the controlled release of the oil and is used in a variety of applications.

Some commonly used techniques for encapsulating oils are emulsification, spray drying, lyophilization, coacervation, and extrusion.^4^ The latter is characterized by its simplicity, low cost and noninvasive effect on the encapsulated material.^7^ The extrusion technique is based on the ionotropic gelation of sodium alginate, where the bioactive ingredient is encapsulated in an alginate solution. During the process, this solution is homogenized and extruded drop by drop, typically using a fine-gauge pipet or syringe, into a solution of calcium chloride, which promotes cross-linking.^8^ The encapsulant is designed to provide greater stability and quality, allowing for controlled release of the components.

Various materials can be used to prepare capsules. The most studied are carbohydrates such as starch, gum arabic, alginate, xanthan and chitosan due to their binding capacity, diversity and low cost.^9^ Sodium alginate is an example of a material widely used for this purpose because it is an anionic biopolymer composed of linear chains of α-l-glucuronic acid and β-d-mannuronic acid. It could form hydrogels, films, spheres, and micro- and nanocapsules in the presence of Ca^2+^ or Mg^2+^ ions.^10^

One approach to innovating the preparation of particles is to incorporate additive materials that enhance or modify their properties, while also modifying the matrices to control release profiles. To achieve this, variations in composition can be employed, such as cross-linking or the addition of functional groups that adjust the size of the particles, as well as the volume or diameter of the pores.^9^ One such material that can be integrated is hydroxyapatite (HA), a calcium phosphate mineral essentially composed of phosphate and calcium that occurs naturally in human bones and teeth, making it highly relevant in bioceramics technology due to its excellent adsorption capacity, biocompatibility, and stability, and is used to replace damaged hard tissue, coat orthopedic prostheses and implants, and repair bone tissue.^11,12^

In a previous study conducted by our research group,^13^ high purity HA was successfully obtained from waste generated during the production of tilapia (*Oreochromis niloticus*) fillets. The produced HA has already been successfully used in biomedical applications, such as the coating of AISI 316L steel for implants^14^ and the fabrication of nanocomposite scaffolds.^15^ The properties of the produced HA show great promise for use as a raw material in the preparation of nanostructured materials. Due to its excellent adsorptive capacity, HA has the potential to adsorb substances such as essential oils, making the particles a promising antimicrobial material.^16^

Essential oils have a wide range of applications in the cosmetic, food, and pharmaceutical industries.^17,18^ One of the essential oils commonly reported for various antibacterial, repellent, and aromatic applications is citronella essential oil.^19,20^ Obtained from the citronella herb (or citronella grass), of the genus *Cymbopogon nardus*, this oil is composed mainly of monoterpenes, including camphene, limonene, 1-borneol, methyl isoeugenol, geranyl, citronellal, citronellol, and geraniol, with the last three being the predominant components. Encapsulating the essential oil protects it from degradation and volatilization and improves its stability and efficacy over time. This technique also allows for the controlled release of active components, which is particularly beneficial in antimicrobial applications.

The combination of HA, sodium alginate, and citronella essential oil provides a set of functional properties that broaden the potential applicability of the resulting particles. The antimicrobial activity of citronella oil, coupled with its controlled release mediated by the alginate matrix, supports their use in applications that require prolonged microbial inhibition, such as active food packaging and antimicrobial wound dressings. Hydroxyapatite, in turn, has been widely studied in polymer-based controlled release systems, particularly in drug delivery, where it acts both as a diffusional regulator and as a structural reinforcement, improving the mechanical stability of the matrix.^21,22^ Moreover, HA has been combined with essential oils in previous studies, with evidence showing enhanced antimicrobial and bone regeneration properties.^16,23^ In this context, the incorporation of HA into the alginate–oil system contributes to structural stability and promotes a more gradual diffusion profile, characteristics that are also desirable for agricultural coatings designed for the controlled release of bioactive compounds.

In this sense, the main objective of this study was to investigate how nanostructured HA, produced from tilapia bones, influences both the antimicrobial activity against *Escherichia coli* and the release behavior of citronella essential oil from alginate-based particles. In parallel, the rheological properties of the emulsions used for particle preparation were evaluated under different HA and alginate proportions to understand their impact on encapsulation capacity. This innovative approach to particle production, using nanostructured HA obtained from animal waste as an adjuvant, represents a promising alternative to conventionally used materials for this purpose. In addition to enhancing the application and efficiency of bioactive encapsulation, this strategy opens new perspectives for the development of more robust and versatile formulations, with the potential to drive significant advances in the field of functional materials, while promoting the sustainable use of biological waste and contributing to the reduction of environmental impact. By integrating complementary functionalities (microbial inhibition, release modulation, and mechanical reinforcement) the proposed particles offer a promising platform for technologies that require sustained delivery of active compounds within biocompatible and environmentally responsible matrices.

## 2 Materials and Methods

### 2.1 Materials

The materials used in this study include pure citronella essential oil (EO) supplied by Aromalife (Brazil), polysorbate 80 surfactant (TWEEN 80, 99%, Oxiteno), USP bidistilled glycerin (99.5%, 21 Química), acetone (CH_3_COCH_3_, P.A., Química Moderna) and calcium chloride (96%, Êxodo). Sodium alginate (NaC_6_H_7_O_6_, Êxodo) used in this study has the following specifications: product code AS04077RA and CAS 9005-38-3. The product appears as a white to beige powder and is composed of α-l-guluronic acid and β-d-mannuronic acid units linked by 1,4-glycosidic bonds.^24^ The product content (concentration) varies between 90.8 and 100%, with low molecular weight (12,000–40,000 g/mol, M/G ratio of 0.8 ^25^). The maximum loss on drying is 15%. The viscosity of a 1% solution at 20 °C is between 300 and 400 cP. The pH is between 6 and 8. In terms of purity, arsenic (As) content is maximum 3 ppm, lead (Pb) up to 5 ppm and heavy metals up to 20 ppm.

The nanostructured HA used in this study was previously synthesized and characterized (see sample A1.3 in Modolon et al.^13^). The phase composition of the nanomaterial was confirmed by X-ray diffraction (XRD), verifying the structural purity of the sample. Only the HA phase (Ca_10_(PO_4_)_6_(OH)_2_) is observed, characterized by hexagonal symmetry and space group *P*6_3_ *m* (ICSD 26204). The most relevant reflections were recorded at 32.55° (300), 33.50° (112), and 40.00° (221), confirming the presence of the typical crystalline structure of HA. More details can be found in the referenced study. To determine the surface area of the HA used in the present work, the Brunauer–Emmett–Teller (BET) method was applied (Quantachrome – NOVA 1200e).

The tilapia bones used in this study were obtained as byproducts from industrial filleting processes intended for human consumption, specifically from Nile tilapia (*Oreochromis niloticus* *)*. No live animals were used at any stage of the research; therefore, ethical approval was not required, in accordance with current Brazilian legislation (Law No. 11,794/2008 – Arouca Law) and the guidelines of the National Council for the Control of Animal Experimentation (CONCEA).^26,27^

### 2.2 Particles Obtention

First, an aqueous HA suspension at a concentration of 10 g/L was prepared by gradually adding HA to 50 mL of deionized water, followed by dispersion for 10 min at 860 rpm under mechanical stirring (Fisatom 713D). The suspension was then subjected to ultrasound treatment for 2 min using a state-of-the-art sonicator (Ultronique), while maintaining constant homogenization by magnetic stirring (Fisatom 752).

To prepare the emulsion that will be converted into particles, sodium alginate, citronella essential oil, Tween 80, glycerin and water were combined in a 250 mL beaker and this mixture was vigorously stirred at 900 rpm for 10 min. After this time, the previously prepared HA suspension was added to the mixture and the resulting emulsion was homogenized for an additional 5 min at 890 rpm. The production procedure is shown in Figure 1.

**Figure 1:**
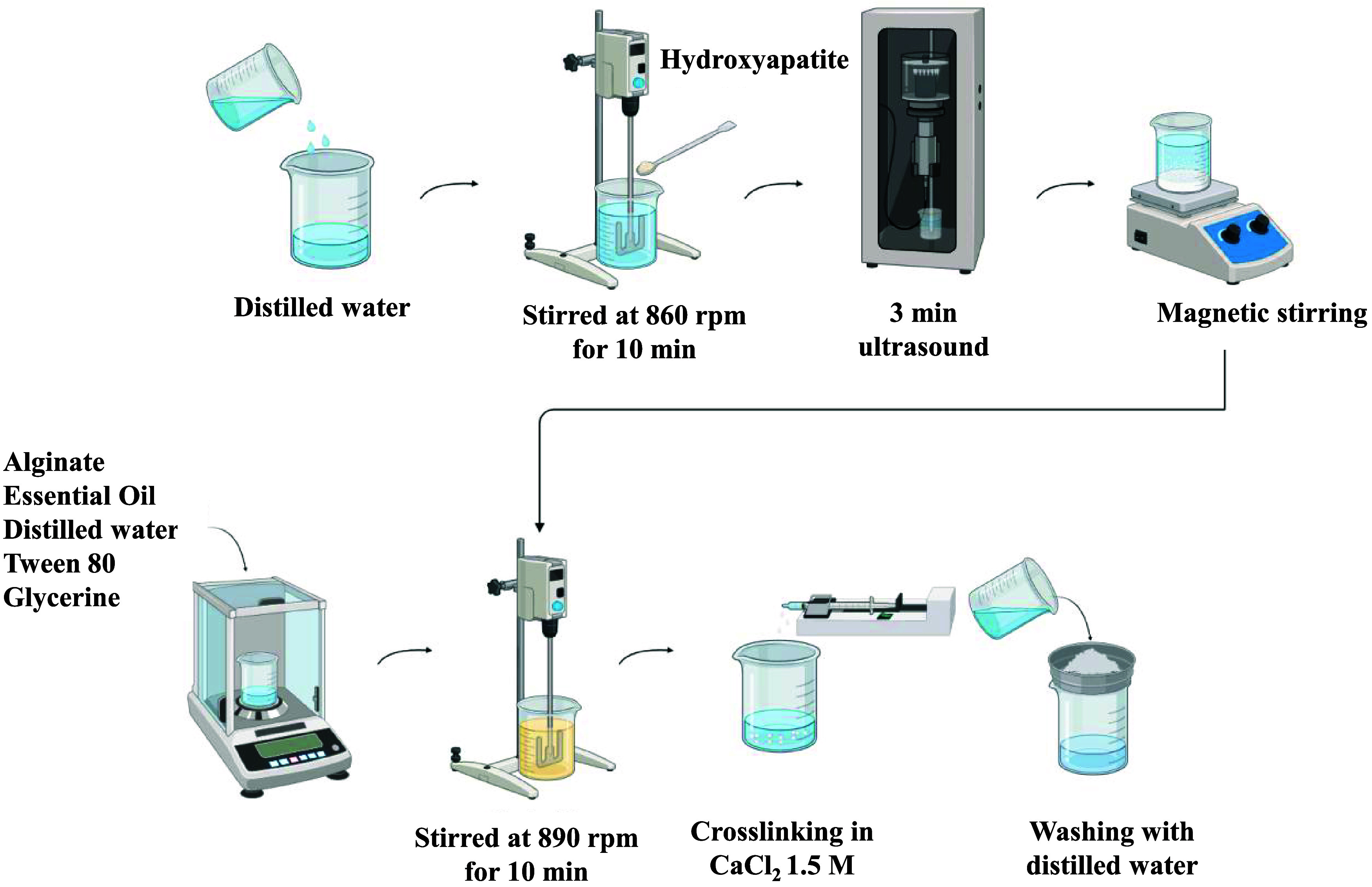
Schematic representation of the particle’s fabrication process.

The compositions tested in this study are presented in Table 1 and include the different proportions of alginate and HA (samples Alg-EO to Alg-EO-H0.15), as well as the proportions used in the preparation of the control samples (samples Pure-Alg and Alg-H0.075). The component values were determined based on the previous study by Lucia de Souza Niero et al.^28^ and on preliminary tests conducted to identify the appropriate proportions of alginate, oil, Tween, and glycerin in the formulation. These tests evaluated the suitable percentage of HA that could be incorporated through ultrasound-assisted suspension and subsequent addition to the emulsion, ensuring a homogeneous dispersion without clogging the syringe.

**Table 1:** Compositions of the Emulsions Prepared for Particle Production

sample	alginate (%)	essential oil (%)	tween (%)	glycerine (%)	HA (%)	water (%)
Alg-EO	2	5.08	2.54	2.5	0	87.88
Alg-EO-H0.04	2	5.08	2.56	2.52	0.04	87.80
Alg-EO-H0.075	2	5.08	2.54	2.54	0.075	87.77
Alg-EO-H0.15	2	5.08	2.5	2.53	0.15	87.74
pure-Alg	2	0	0	0	0	98
Alg-H0.075	2.09	0	2.69	2.67	0.079	92.53

The rheological behavior of the emulsions was evaluated using a Thermo Scientific HAAKE MARS IQ rotational rheometer equipped with a double cone/plate sensor configuration, requiring a sample volume of 1–3 mL. The measurement process involved three velocity (shear) mode steps at a constant temperature of 25 °C. In the first step, the shear rate was linearly increased from 0 to 400 s^–1^ over 300 s. The second step maintained a shear rate of 400 s^–1^ over 120 s, followed by a third step where the shear rate was linearly decreased from 400 to 0 s^–1^ over 300 s. Flow curves were then generated to evaluate emulsion stability, thixotropy, and viscosity.

Finally, the emulsions were dripped into a 1.5 M CaCl_2_ solution using a syringe pump (DBM Eletrotech) with a 22G needle (0.70 mm orifice) at a flow rate of 0.25 mL/min, where they were left for 30 min. After this period, the samples were rinsed with distilled water, transferred to a watch glass, and placed in a desiccator for 24 h.

### 2.3 Particle Characterization

Particle analysis was performed using the ImageJ software, based on images acquired using a Bioptika L60 (stereoscopic microscope) at a total magnification of 8×. Each image included a visible scale corresponding to a micrometric ruler previously calibrated in the optical system. To ensure reproducibility, the “Set Scale” function in ImageJ was applied to establish the relationship between image pixels and actual micrometer units, using the scale present in each image. Measurements were manually conducted using the “Straight Line” tool to trace the diameter of the particles. Each sample was evaluated in three independent replicates, with three distinct measurements per replicate, totaling nine measurements per sample.

Detailed examination of particle morphology was performed using a scanning electron microscope (SEM, EVO MA10, Zeiss). For microstructural analysis, samples of the fractured surfaces were coated with a thin gold (Au) layer prior to SEM imaging.

Porosity was estimated by calculating the density of the particles. True density was measured using helium gas pycnometry (Anton Paar, Ultrapyc 5000). These data, together with the apparent density (ρ_ap_) of the sample (determined by the mass-to-volume ratio, where mass was measured using an analytical balance and volume was calculated from size measurements using a stereoscopic microscope), allowed the relative density and hence the porosity of the particles to be calculated.

Fourier transform infrared spectroscopy (FTIR, Tensor II model) was used to identify the vibrational modes of chemical bonding groups in both the particles and the essential oil. Spectra were obtained in the absorption mode, covering a range from 4000 to 400 cm^–1^. This analysis is crucial for identifying functional groups and characterizing the chemical composition of the compounds present within the particle structure.

### 2.4 Antimicrobial Assay

The antimicrobial potential of the particles was evaluated using the agar diffusion method with *Escherichia coli* (Gram-negative) bacteria according to the protocol described by Bauer et al.^29^ All materials used in the test were sterilized at 121 °C for 15 min in a vertical autoclave (Phoenix). For the assay, the solid culture medium was prepared by dissolving 38 g of Mueller-Hinton agar in 1 L of distilled water, while the liquid culture medium was prepared by dissolving 3.70 g of BHI broth in 100 mL of distilled water.

To ensure the purity of the sample, the microorganism was inoculated on Petri dishes containing solid culture medium. After 24 h of incubation, a single colony forming unit (CFU) of the bacterium identified as pure was isolated and transferred to a glass tube containing 5 mL of liquid culture medium. The tube was then incubated at 37 °C for 24 h in an oven (FANEM).

The microorganism was then diluted 1:10 in a tube containing 9 mL of 0.9% saline. The diluted bacteria were then inoculated onto Petri dishes using an inoculation loop. To evaluate the inhibitory potential of the samples, 0.8 g of particles were added to each plate, which was calculated to contain a sufficient amount of essential oil to inhibit bacterial growth. The assay was performed in duplicate and all of the samples listed in Table 1 as well as pure HA were evaluated. The plates were incubated 24 h at 37 °C in an oven (FANEM). After incubation, the diameter and area of the inhibition halo around each sample were measured to evaluate the antimicrobial potential of the samples.

### 2.5 Release Kinetics of Essential Oil

To evaluate the release kinetics of essential oil from the particles, 0.8 g samples were exposed to the environment at 25 °C. At specific time intervals (ranging from 0 to 96 h), the samples were transferred into 10 mL of acetone to extract the remaining essential oil within the particles. The mixture was then homogenized for 24 h on a shaking table (Quimis, model Q225MT). The test was performed in triplicate. After extraction, the particles were separated from the acetone by decantation, and the concentration of residual essential oil in the liquid phase was analyzed using a UV–vis spectrophotometer (Shimadzu, model UV 1800). For this, a calibration curve was constructed following the methodology described by Bezerra et al.,^30^ using the dilutions of the essential oil in acetone and the areas of the absorbance curves obtained during scanning in the range of 250 to 550 nm. This curve was used to quantify the essential oil in each experiment carried out.

Encapsulation efficiency was determined according to the methodology described by Pratiwi et al.^31,32^ For this purpose, an aliquot of 0.1 g of the emulsion, prior to the encapsulation process, was mixed with 10 mL of acetone and kept under agitation on a shaker table for 24 h. The sample was then analyzed by UV–vis spectrophotometry, and this value was considered as the maximum reference of oil present before encapsulation. After the encapsulation process and washing of the particles with distilled water, 0.1 g of particles were macerated and again mixed with 10 mL of acetone, subjected to homogenization, and analyzed by UV–vis. This procedure allowed quantification of the fraction of oil retained in the particles. Encapsulation efficiency was calculated as the ratio between the amount of oil present in the particles after encapsulation and the initial amount of oil in the emulsion, multiplied by 100.

The sample that showed the best performance in the antimicrobial assay was selected to study the residual essential oil profile in the particles over time. For this purpose, 0.2 g of the selected sample was placed in an enclosed space at 25 °C. At set time intervals ranging from 0 to 180 min, the sample was transferred to 4 mL of acetone to extract the oil retained in the matrix. The assay was performed in duplicate. After immersion in acetone, the samples were homogenized for 24 h. Then, the particles were separated from the solvent, and the acetone was analyzed by UV–vis spectrophotometry to quantify the extracted oil. Statistical analysis was performed using GraphPad Prism (version 8). Data are presented as mean ± standard deviation. For all analyses, *p* < 0.05 was considered statistically significant. Comparisons were evaluated using two-way analysis of variance (ANOVA).

## 3 Results and Discussion

### 3.1 Characterization of Nanostructured Hydroxyapatite Derived from Tilapia Bones

The surface properties of HA were determined using the Brunauer–Emmett–Teller (BET) method, and the results are presented in Figure 2. The nanostructured material has a specific surface area of 31.319 m^2^/g and an average particle diameter of 87.081 nm. The material is classified as mesoporous, which can be attributed to two main reasons: first, the pore size distribution, shown in Figure 2a, indicates an average pore diameter of 13.584 nm, which falls within the characteristic range of mesopores, between 2 and 50 nm;^33^ second, the adsorption isotherm, also presented in Figure 2b, exhibits a hysteresis pattern typical of type IV isotherms, associated with mesoporous materials. This hysteresis behavior arises from the difference between gas adsorption and desorption in the pores, confirming the presence of intermediate-sized pores.^34^

**Figure 2:**
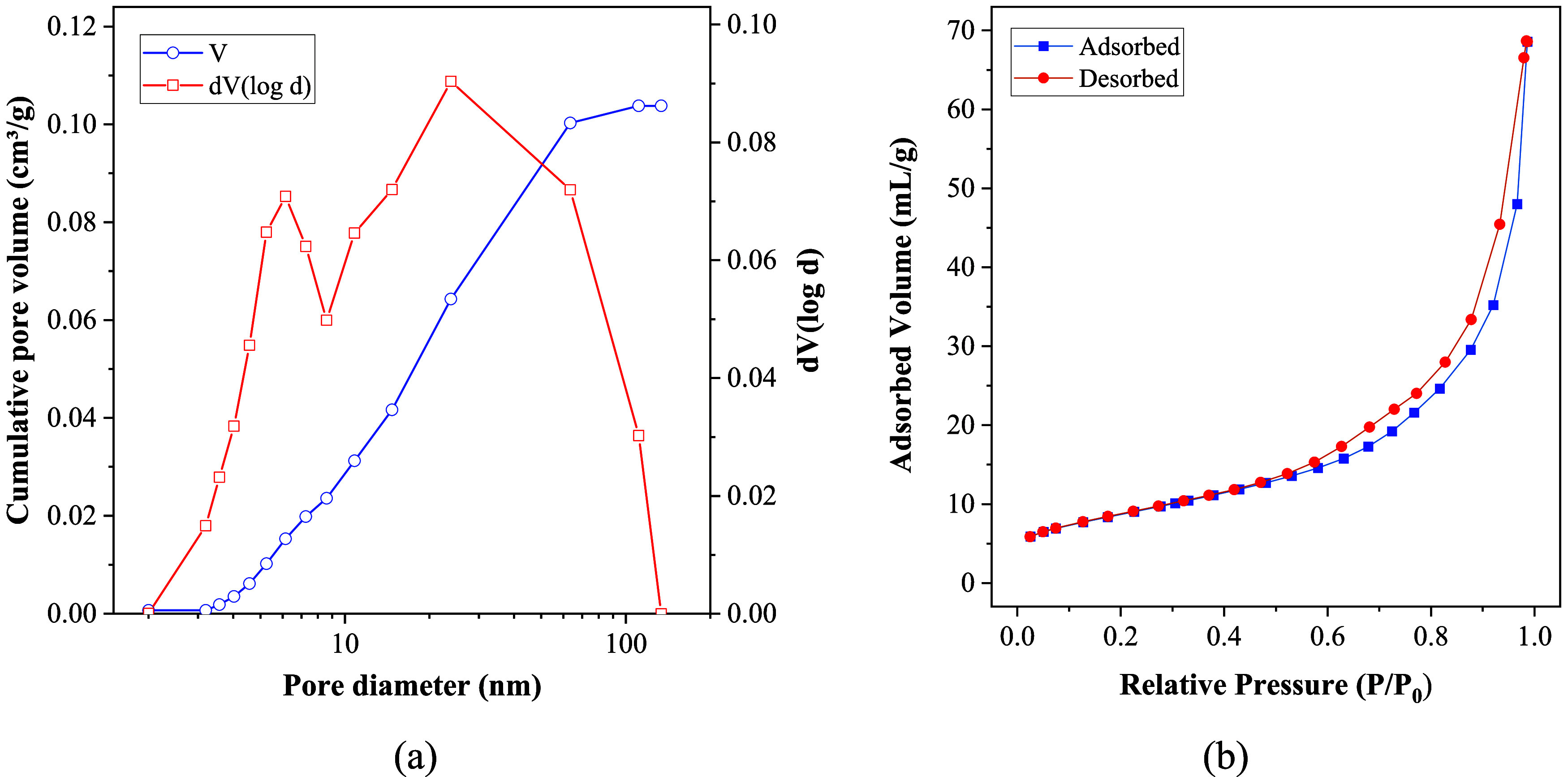
Pore volume distribution (a), adsorption and desorption isotherms of hydroxyapatite derived from tilapia (*Oreochromis niloticus*) bones (b).

The mesoporous structure is advantageous for the loading of bioactive materials, as it provides a larger surface area and an extensive pore network that facilitates the incorporation and retention of active components within its matrix.^35^ Mesoporous HA has been investigated as a vehicle for the controlled delivery of bioactive materials, with objectives similar to those of the present study. For example, Li et al.^35^ employed it for the release of doxorubicin hydrochloride in cancer treatment, while Lin et al.^36,37^ used it for the release of clenbuterol in the treatment of Alzheimer’s disease.

### 3.2 Rheological Evaluation

The introduction of HA into the emulsion represents the incorporation of solids into the matrix, which can have a significant impact on the rheology of the system. The addition of solids can alter the viscosity and thixotropy of the emulsion, directly affecting its practical applicability. Understanding how the ratio of these solids affects the rheological properties is critical to ensuring emulsion stability, controlling drug release, and optimizing the particle manufacturing process. The flow curves for the samples analyzed are shown in Figure 3a.

**Figure 3:**
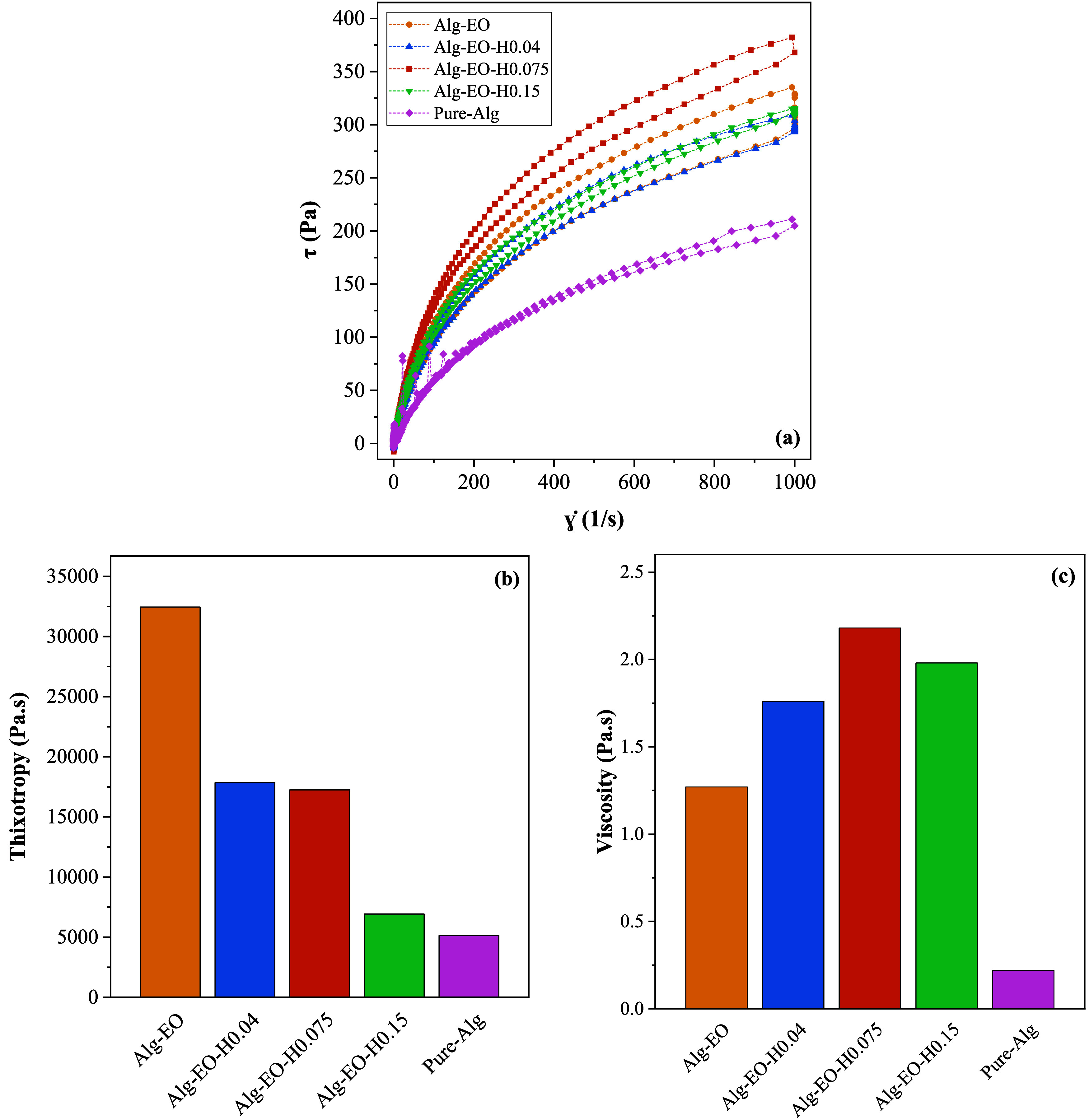
Flow curves (a), thixotropic behavior (b), and viscosity profiles (c) of the emulsion’s samples.

In general, it is clear from the flow curves that there is no evidence of agglomeration of HA or alginate particles. This suggests that the dispersion with ultrasound and magnetic stirring was effective in breaking up weak agglomerates between the particles. The sample containing only alginate (sample Pure-Alg) has the lowest flow resistance, as expected, since the introduction of particles in such a system tends to increase the flow resistance, especially at high shear rates.^38,39^

The curves show that all the emulsions studied exhibit pseudoplastic behavior, characterized by a decrease in viscosity with fluid deformation. In addition, all the emulsions showed thixotropic properties. According to Corrêa et al.,^39,40^ the thixotropic nature of emulsions is advantageous because it results in increased fluidity over time, which prevents the product from running and facilitates application. In this case, such behavior aids in the particles encapsulation process, allowing them to retain their shape after dropping into the cross-linker.

The thixotropy and viscosity values for each sample are shown in Figure 3b,c, respectively. The sample without HA (sample Alg-EO) has the highest thixotropy of all the samples analyzed, with a value of 32,450 Pa.s. Furthermore, an increase in HA concentration corresponds to a decrease in thixotropy. The sample with the highest HA content (Sample Alg-EO-H0.15) shows a thixotropy similar to that of the alginate-only sample, indicating that both formulations have a greater tendency to flow.

Viscosity also plays a very important role in particle processing as it significantly affects the stability of the emulsion over time, especially in the presence of solid components such as HA. The viscosity values obtained during the particle dropping process were determined at the same shear rate used during the syringe dropping process, reflecting the real conditions of the particle manufacturing process. Increasing the amount of HA results in higher viscosity as the solids content of the mixture is increased.^39^ This is interesting because increasing the viscosity of the emulsion impedes circulation within the droplets and therefore results in rapid formation of the particle wall.

The sample with the highest HA concentration (sample Alg-EO-H0.15) showed a decrease in viscosity compared to the sample with a lower HA content (sample Alg-EO-H0.075). This observation suggests the presence of a saturation point beyond which an increase in solid content may compromise the stability of the emulsion and negatively affect the encapsulation process. This phenomenon could be attributed to an excessive fluidity of the wall material, which could lead to a nonuniform distribution of the active components during the formation of the microspheres.^41,42^ With this result, sample Alg-EO-H0.075 appears to be the most suitable for the particle dropping process, as it has the ideal viscosity to ensure stable formation of encapsulated particles.

### 3.3 Particle Characterization

Scanning electron microscopy (SEM) analyses were performed to evaluate the morphology and microstructure of the particles, as shown in Figure 4. The samples exhibited irregular surface textures and a continuous internal structure, with no visible pores detected on the particle surfaces. A predominantly spherical or semispherical morphology was observed across all formulations, with a robust internal architecture. Magnified cross-sectional views of particles Alg-EO-H0.075 and Alg-EO-H0.15 can be found in the Supporting Information (Figure S1).

**Figure 4:**
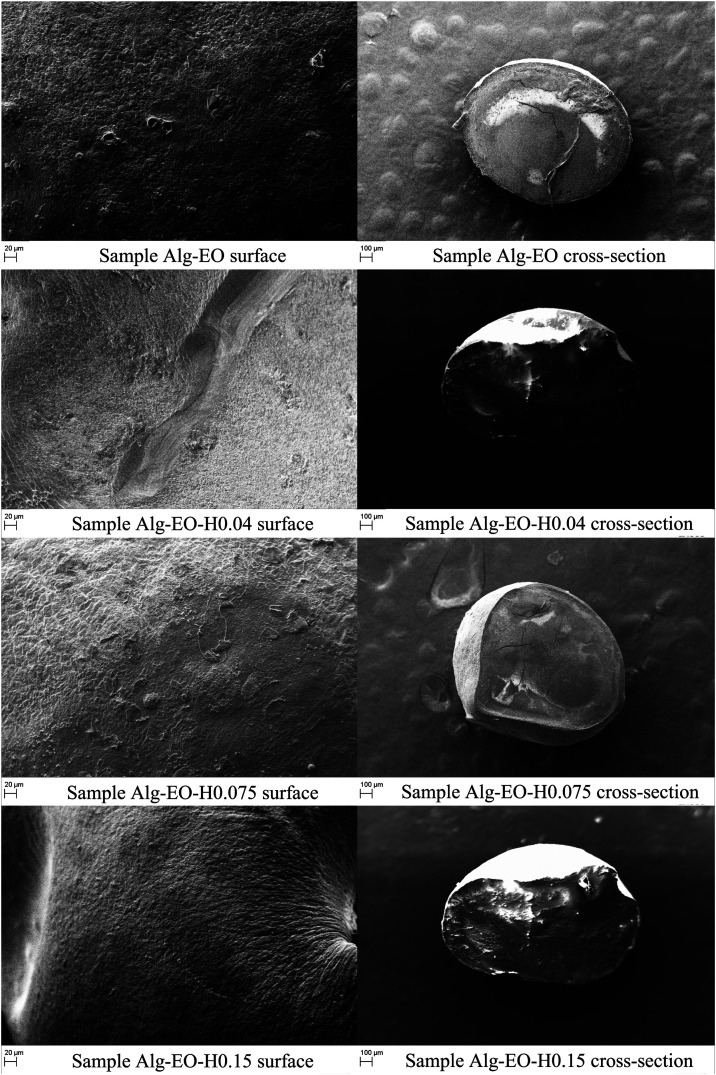
Particle surface (500× magnification) and cross-section (100× magnification) micrographs.

The measured porosities (Table 2) were 25.0 ± 4.19 for Alg-EO, 27.3 ± 6.40 for Alg-EO-H0.04, 24.0 ± 9.68 for Alg-EO-H0.075, and 28.9 ± 5.26 for Alg-EO-H0.15, indicating that, despite numerical variation, the increase in HA concentration did not result in statistically significant differences in particle porosity. In comparison, Niero and Arcaro^28^ reported substantially higher porosities, ranging from 69 to 78%, in Alg/HA (1:15) formulations. The discrepancy between the studies can be attributed primarily to the higher amount of HA employed in their work, whereas the formulations developed here used lower concentrations, which explains the reduced porosity values and the absence of pores along the particle surfaces observed by SEM.

**Table 2:** Density and Porosity of the Samples Analyzed

sample	real density (g/cm^3^)	apparent density (g/cm^3^)	porosity (%)
Alg-EO	1.56 ± 0.01	1.15 ± 0.06	25.0 ± 4.19
Alg-EO-H0.04	1.58 ± 0.02	1.11 ± 0.09	27.3 ± 6.40
Alg-EO-H0.075	1.54 ± 0.02	1.17 ± 0.14	24.0 ± 9.68
Alg-EO-H0.15	1.53 ± 0.03	1.10 ± 0.08	28.9 ± 5.26

The statistical analysis (two-way ANOVA) revealed a significant effect only for the factor related to the type of physical property, indicating that bulk density, relative density, and porosity differ significantly from one another. On the other hand, no significant differences were observed among the hydroxyapatite (HA) concentrations, nor in the interaction between the factors, reinforcing that the variation in HA concentration in this study did not have a relevant impact on these physical properties. The detailed statistical values are provided in Supporting Information (Table S1).

The mean diameters of the particles in the samples ranged from 2.567 to 2.810 mm (Figure 5). Sample Alg-EO-H0.04 presented the largest mean diameter (2.810 ± 0.052 mm), while sample Alg-EO-H0.075 presented the smallest mean diameter after preparation (2.567 ± 0.077 mm). This result can be explained by the higher viscosity of the emulsion in sample Alg-EO-H0.075, which favored the formation of smaller and more uniform particles. This occurs because increased viscosity suppresses internal circulation movements within the droplets, promoting faster solidification of the particle wall.^39^ In contrast, the lower viscosity of the emulsion in sample Alg-EO-H0.04 resulted in less efficient particle formation, leading to the production of particles with larger diameters.

**Figure 5:**
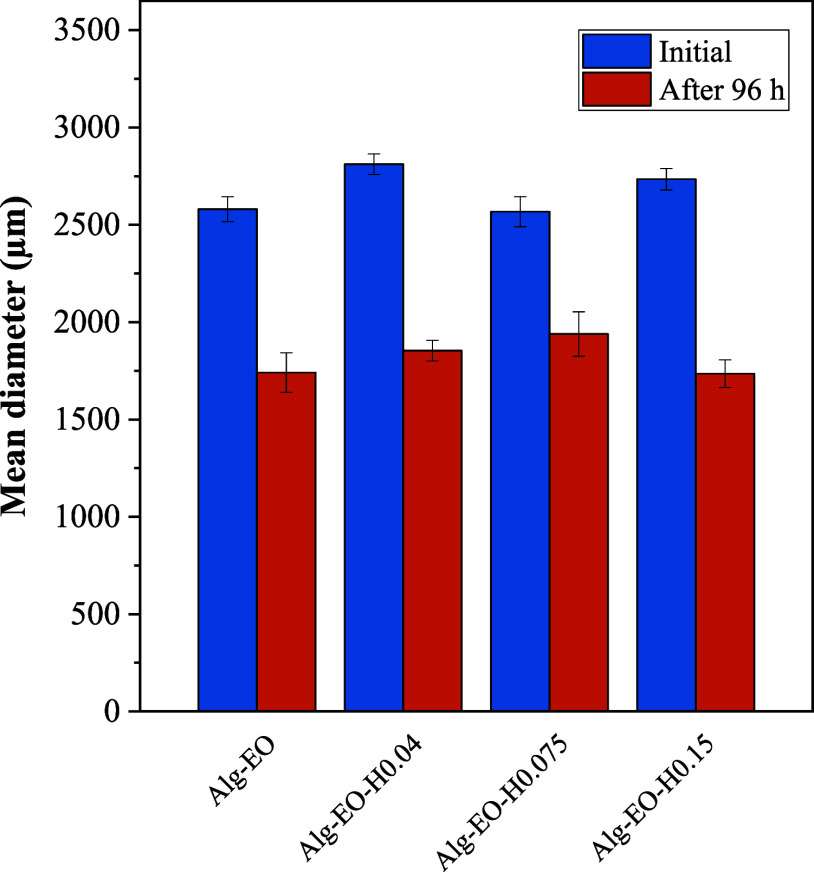
Mean particle diameter, initial and after 96 h.

All samples showed a reduction in size after 96 h exposure, with a reduction in diameter of approximately 30% for samples Alg-EO, Alg-EO-H0.04 and Alg-EO-H0.15. It is noteworthy that sample Alg-EO-H0.075, which initially had the smallest mean diameter after preparation, showed the smallest reduction in diameter over the exposure period, with a reduction of only 23.6%. This reduction is associated with the loss of mass, mainly water and essential oil, to the environment. The rate of loss of essential oil and water is influenced by the structure of the particles and their ability to retain these components over time. The lower loss and consequent reduction in diameter observed in sample Alg-EO-H0.075 is corroborated by the results of the rheological analysis of the emulsions, which showed that this sample reached an optimum point without exceeding the dispersion capacity of the wall material. This characteristic has a direct impact on the effectiveness of the encapsulation and, consequently, on the ability of the sample to store the essential oil and the water of the composition for a prolonged exposition period.

The statistical analysis (two-way ANOVA) revealed significant effects for all evaluated factors: hydroxyapatite (HA) concentration, exposure time, and the interaction between these factors. These results demonstrate that particle diameter was significantly altered over time, that different HA concentrations strongly influenced the magnitude of the observed reduction, and that the effect of concentration was time-dependent, varying according to the exposure period considered. The detailed statistical values are provided in Supporting Information (Table S2).

The FTIR spectra (Figure 6) show that all samples have similar spectral characteristics, with the same characteristic bands. The O–H group was identified in the broad bands close to 3300 cm^–1^, mainly related to the water content. Except for the sample Alg-EO (without HA), doublets were also observed in the bands between 1000 and 1100 cm^–1^, corresponding to the PO_4_ groups of HA. The characteristic C=O band originating from sodium alginate at about 1630 cm^–1^ was identified in all samples.^43,44^ The band around 1422 cm^–1^ is attributed to C–O stretching present in alginate. Furthermore, the intensity of the PO_4_ peak increased with higher HA concentrations. Finally, sample Alg-H0.075, which does not contain essential oil in its composition, did not show any other different peak in its spectra, indicating that the oil likely does not adhere to the surface of the particles.

**Figure 6:**
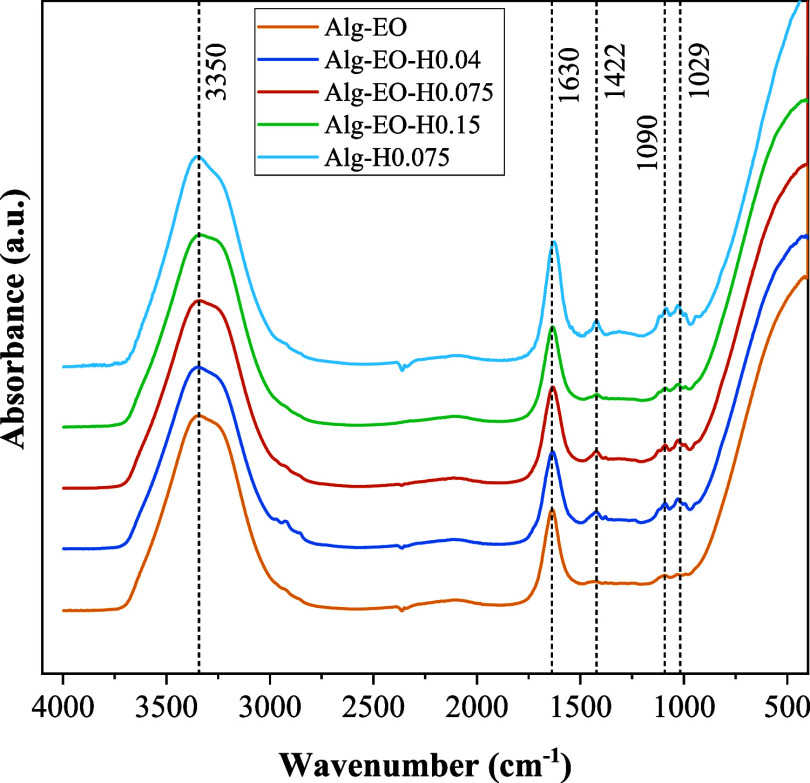
FTIR spectra of the particle samples.

### 3.4 Antimicrobial Test

The results of the agar diffusion test for the analyzed samples (Alg-EO to Alg-H0.075 and pure HA) are shown in Figure 7. The mean diameters of the inhibition halos and the areas for each sample are provided in Table 3.

**Figure 7:**
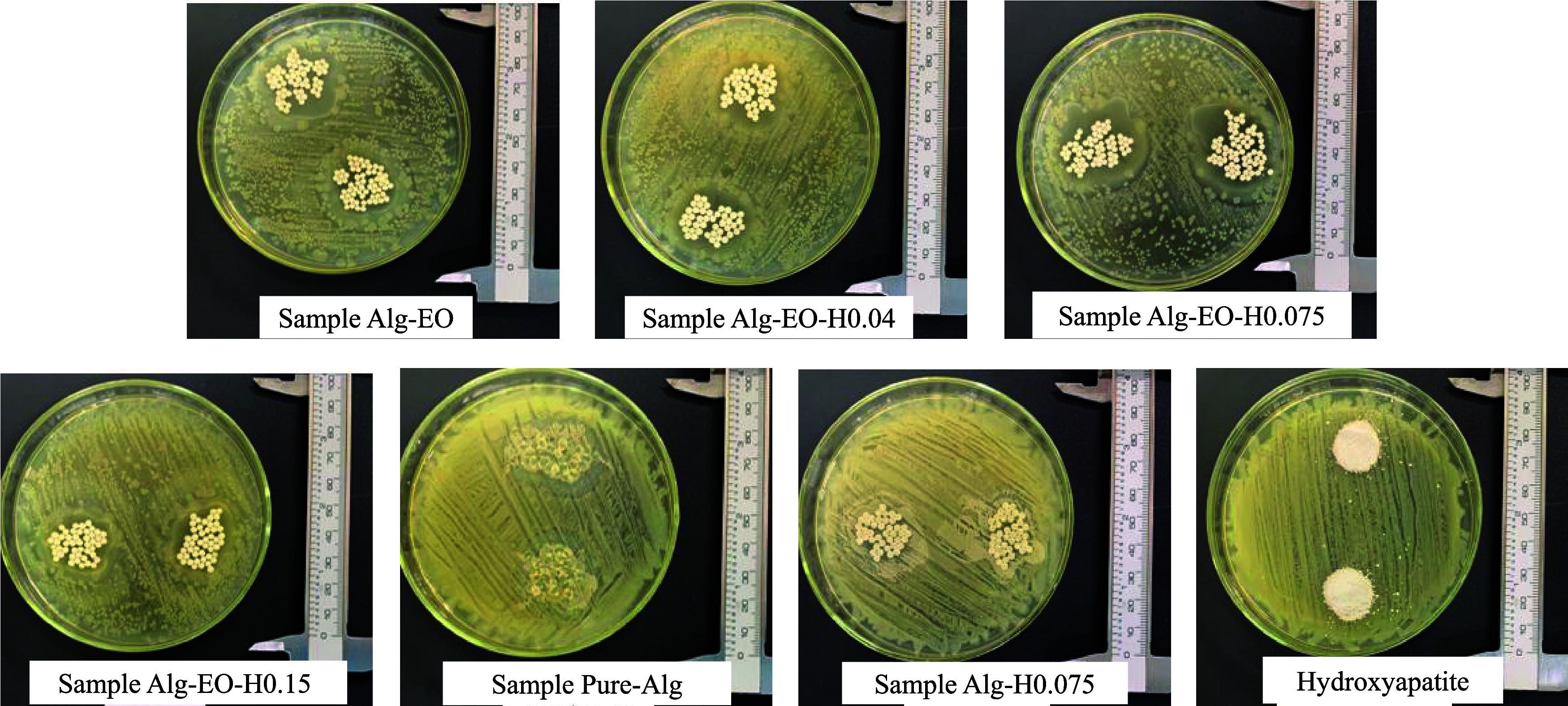
Inhibition halos of the particle samples and pure hydroxyapatite.

**Table 3:** Mean Diameter and Area of Inhibition Halos

sample	mean diameter (mm)	area (cm^2^)
Alg-EO	30 ± 2	6.73
Alg-EO-H0.04	27 ± 2	5.67
Alg-EO-H0.075	34 ± 3	8.40
Alg-EO-H0.15	26 ± 2	5.41
pure-Alg	without halo	
Alg-H0.075	without halo	
pure hydroxyapatite	without halo	

The samples containing essential oil exhibited inhibition halos around the application points, indicating their ability to inhibit microbial growth. The area and mean diameter of the halos varied according to the formulation of the particles, with sample Alg-EO-H0.075, which contained an intermediate percentage of HA, demonstrating the greatest antimicrobial potential, with a mean diameter of 3.4 ± 0.3 cm and an area of 8.4 cm^2^. It is important to highlight that the particles formulated only with alginate and water (sample Pure-Alg), as well as those without essential oil (sample Alg-H0.075) and pure HA, did not show any inhibition halo. This confirms that the antimicrobial efficacy is directly related to the presence of the essential oil, and that the incorporation of HA enhanced this effect.

Sample Alg-EO-H0.075 exhibited an oil concentration of 4.0 mg/L, determined from the immediate extraction of encapsulated oil (without exposure time) using 10 mL of acetone and 0.8 g of particles, the same amount used per plate in the antimicrobial assay. Quantification was performed through the release assay described in Section 2.5, using a calibration curve. This concentration corresponds to an oil mass of 0.040 mg (calculated by multiplying the concentration by the acetone volume) and a volume of 44.5 nL, based on the oil density of 897.2 kg/m^3^. In previous studies, Brugnera et al.^45^ observed an inhibition diameter of 3.42 ± 0.35 mm with 0.3125 μL per plate of citronella essential oil, while El Kamari et al.^46^ achieved a diameter of 18.0 ± 0.5 mm with Only 15 mg of oil, which corresponds to a volume of 16.72 μL. The results agree with previous studies but demonstrate greater efficiency, as larger inhibition halos were obtained with smaller oil volumes.

Indeed, the agar diffusion test showed that the particles encapsulated with citronella essential oil have significant antimicrobial potential. Among the formulations, the one with an intermediate concentration of HA (sample Alg-EO-H0.075) proved to be the most effective in inhibiting microbial growth. These results highlight the potential of these particle formulations for the development of new antimicrobial solutions for *E. coli* as well as other bacteria, in various medical and industrial contexts.

Previous studies show that citronella essential oil has antimicrobial potential capable of inhibiting various bacteria beyond *E. coli*. El Kamari et al.^47^ reported inhibitory effects against various bacteria, including Gram-positive species such as *S. aureus* and *E. faecalis*, as well as Gram-negative species like *K. pneumoniae* and *P. aeruginosa*. Wei and Wee^48^ validated the inhibitory action of citronella essential oil against systemic bacteria isolated from different aquatic animals, such as *Aeromonas spp., Edwardsiella spp.*, and *Flavobacterium spp.*, which showed the highest inhibition potentials. The results obtained with *E. coli* demonstrate the antimicrobial potential of the particles developed in this study. Considering that citronella essential oil has been widely described in the literature as effective against various Gram-positive and Gram-negative bacteria, it is plausible to assume that these particles may also exhibit activity against other microorganisms sensitive to citronella oil.

### 3.5 Release Kinetics of Essential Oil

The objective of the developed particles is to achieve a controlled and sustained EO release into the air over time. In this context, the *C*/*C*_*t*_0__ profiles provide indirect information on the oil volatilization behavior by quantifying the residual oil retained in the particles after exposure to air for different periods (Figure 8). Lower *C*/*C*_*t*_0__ values indicate a higher loss of oil to the atmosphere, whereas higher values indicate stronger retention within the porous matrix. Thus, the different trends observed among the formulations reflect the balance between oil–matrix interactions, internal diffusion within the porous structure, and volatilization to the surrounding air. According to the literature, 24 h are sufficient to evaluate antimicrobial activity. In this study, however, the analysis period was extended to 96 h in order to enable both the assessment of antimicrobial activity and the acquisition of a more consistent release profile over a prolonged interval.^49,50^

**Figure 8:**
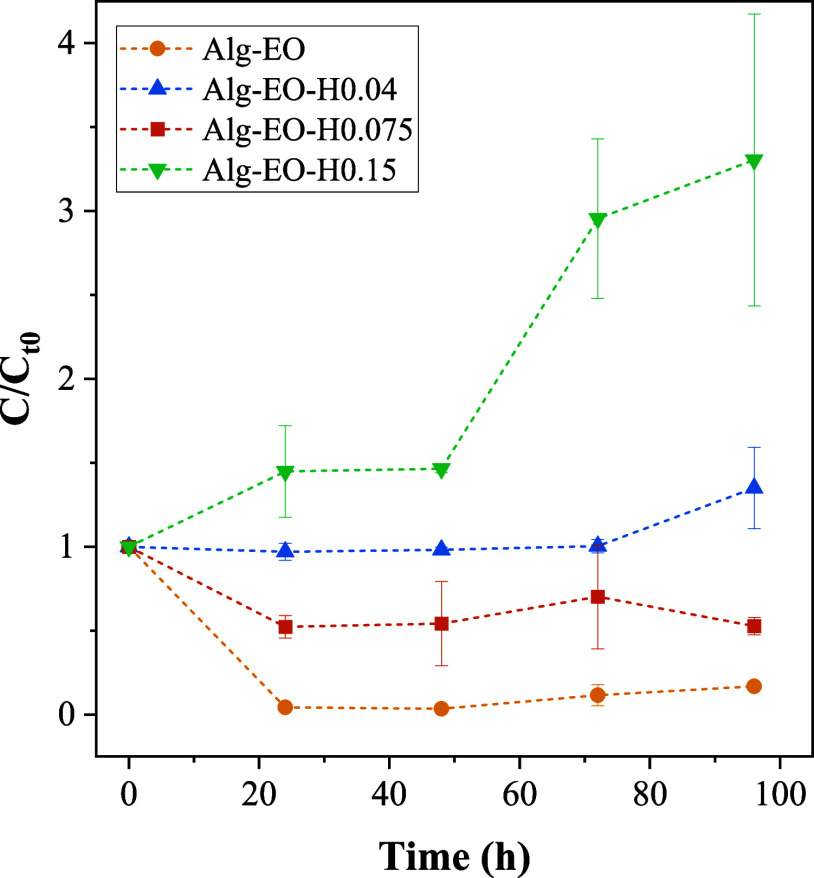
Citronella essential oil release in acetone as a function of time.

The Alg-EO particles exhibited the lowest *C*/*C*_*t*_0__ values throughout the experiment, indicating that a significant fraction of the essential oil was released to the air during exposure. This suggests that the alginate-only matrix provides weak oil retention, allowing the oil to diffuse easily through the porous network and volatilize. The Alg-EO-H0.04 formulation exhibited a stable *C*/*C*_*t*_0__values, indicating moderate retention of the essential oil and a gradual loss to the air over time.

A similar behavior was observed for the Alg-EO-H0.075 sample, which showed a decrease in the *C*/*C*_*t*_0__ ratio at early times followed by stabilization, suggesting an initial release of more weakly bound or surface-associated oil, followed by a slower release of the fraction retained within the internal structure during the first 24 h. This behavior is consistent with the rheological characteristics of these formulations, with particular emphasis on Alg-EO-H0.075, which exhibited the most suitable viscosity for particle formation and rapid wall development. In combination with the observed release behavior, this formulation provides an effective balance between initial oil availability and controlled release, which is advantageous for practical applications. A release profile with a more constant rate is important to ensure that the amount of active ingredient available in the environment is released gradually, avoiding an abrupt release of the entire content.^51^

In contrast, the Alg-EO-H0.15 sample exhibited increasing *C*/*C*_*t*_0__ values over time, indicating strong retention and minimal loss of essential oil to the air. Such excessive retention may hinder the intended controlled release, limiting its applicability to systems that require continuous emission of oil into the environment. This behavior can be attributed to the fact that Sample Alg-EO-H0.15 contains a solid concentration above the solubility limit of the polymer.^52^ This profile is supported by the significant decrease in viscosity observed with increasing HA, as revealed by the rheological tests.

The statistical analysis (two-way ANOVA) revealed significant effects for both main factors and their interaction: release time, hydroxyapatite (HA) concentration, and the interaction between time and concentration. These results demonstrate that the amount of essential oil released increases progressively over time, that different HA concentrations significantly affect the release, and that the impact of concentration is time-dependent, varying according to the release interval considered. The detailed statistical values are provided in Supporting Information (Table S3).

The encapsulation efficiency obtained for sample Alg-EO-H0.075, which showed the best antimicrobial performance, was 38.37% ± 3.04. This value is consistent with the range reported in the literature for essential oil encapsulation, where different studies describe efficiencies that vary considerably, from lower values such as 5.7 and 21% to higher results approaching 60% and even 97%, depending on the production method and experimental conditions employed.^53–55^

A complementary study was carried out to evaluate the residual essential oil concentration in the particles as a function of exposure time in the environment. This analysis was conducted with the Alg-EO-H0.075 sample, selected for presenting a controlled kinetic profile and, at the same time, superior performance in the rheological and antimicrobial assays. The results are shown in Figure 9.

**Figure 9:**
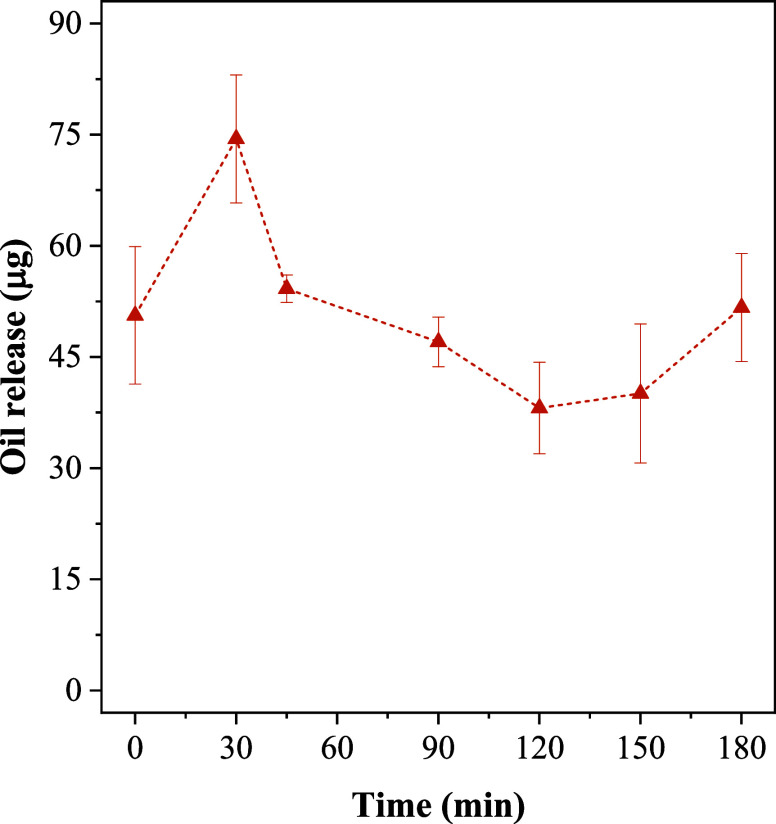
Residual oil concentration as a function of exposure time to the environment.

It was expected that the amount of residual oil would continuously decrease over time. However, the obtained release profile exhibited fluctuations in the measured concentrations, suggesting that the release process did not follow a Fickian kinetic model, in which the mass flux is directly proportional to the concentration gradient. Furthermore, the experimental data did not fit adequately to any of the evaluated kinetic release models. This anomalous behavior indicates a heterogeneous distribution of the essential oil within the particles, resulting in irregular release patterns over time. Such heterogeneity, already reported in the literature, may arise from intrinsic structural variations within the particles, which promote different release rates at short time intervals.^56^ This hypothesis was supported by duplicate analyses using samples from the same batch and identical mass, which yielded distinct amounts of extracted.

Although not intentionally designed, the observed heterogeneity may represent an advantage for multiphasic applications, allowing different fractions of the essential oil to be released at distinct times and thereby prolonging its action. Previous studies have demonstrated that systems with multiple phases or layers favor this type of controlled release. Moshe et al.^57^ showed that the selective distribution of thymol among polymeric phases resulted in distinct release profiles, while Zhang et al.^58^ employed double-layered microcapsules to modulate the release of lavender oil. These findings reinforce that the presence of structural heterogeneity, even when unintended, can be explored as a promising approach to optimize the efficacy of bioactive compounds.

## 4 Conclusions

In conclusion, this study successfully demonstrated the encapsulation of citronella essential oil in sodium alginate particles containing HA derived from tilapia bones, highlighting the influence of HA addition on the structural characteristics, controlled oil release, and antimicrobial activity against *E. coli* of the particles. The incorporation of HA directly affected the rheological behavior of the emulsions, with higher concentrations increasing viscosity and reducing thixotropy, which influenced the formation and stability of the particles. Among the samples tested, sample Alg-EO-H0.075 presented the ideal HA concentration, with a balance between viscosity and thixotropy that favored the formation of stable particles with good encapsulation efficiency.

The release kinetics showed that the HA-containing samples exhibited more gradual citronella release profiles over time. The Alg-EO-H0.15 sample, with the highest HA content, initially retained more essential oil but subsequently displayed an abrupt release, compromising the controlled profile. In contrast, the Alg-EO sample (without HA) released almost all of the oil within the first 24 h, confirming its low retention capacity. The Alg-EO-H0.04 and Alg-EO-H0.075 samples presented more controlled and comparable release rates, with Alg-EO-H0.075 standing out due to its adequate kinetic behavior and superior rheological and antimicrobial performance, confirming its overall higher efficiency.

Antimicrobial assays confirmed the efficacy of encapsulated citronella essential oil in inhibiting the growth of *E. coli*, demonstrating the potential of these particles for antimicrobial applications against other pathogenic species. Furthermore, the presence of HA was found to enhance the antimicrobial effect of the essential oil, promoting its diffusion and prolonging its action. Thus, HA acted not only as a structural additive but also as a functional agent, contributing to the stability, retention, and bioactive performance of the particles. These results indicate that the particles present characteristics that are compatible with potential applications such as antimicrobial wound dressings and active food packaging, which benefit from sustained antimicrobial activity. In addition, the developed formulations demonstrate versatility that may allow the incorporation of different bioactive agents and, if further optimized, could be tailored into different formats for specific controlled-release purposes. Such prospects expand the possible applicability of these materials in areas such as healthcare, agriculture, and cosmetics, provided that future studies validate their performance under conditions representative of each intended use.

## Supplementary Material



## Data Availability

The data supporting this study are available within the manuscript and the Supporting Information file.

## Notes

Cross-sectional micrographs of particles (Figure S1), detailed two-way ANOVA statistical results for physical properties (Table S1), particle diameter variations (Table S2), and essential oil release kinetics (Table S3). This Supporting Information offers extended characterization and statistical analysis supporting the findings discussed in the manuscript (PDF)

## References

[1] Macedo D. F., Dourado S. M., Nunes E. S., Marques R. P., Moreto J. A. (2019). Controlled
Release of TBH Herbicide Encapsulated on Ca-ALG Microparticles: Leaching
and Phytointoxication Plants. Planta Daninha.

[2] Lins L., Dal Maso S., Foncoux B., Kamili A., Laurin Y., Genva M., Jijakli M. H., De Clerck C., Fauconnier M. L., Deleu M. (2019). Insights into the Relationships between
Herbicide Activities, Molecular Structure and Membrane Interaction
of Cinnamon and Citronella Essential Oils Components. Int. J. Mol. Sci..

[3] Wang L. (2024). Preparation
and Characterization of Natural Fragrant Microcapsules. J. Fiber Bioeng. Inf..

[4] Bakry A. M., Abbas S., Ali B., Majeed H., Abouelwafa M. Y., Mousa A., Liang L. (2016). Microencapsulation
of Oils: A Comprehensive
Review of Benefits, Techniques, and Applications. Compr. Rev. Food Sci. Food Saf..

[5] Pereira K. C., Mota Ferreira D. C., Alvarenga G. F., Salvador Pereira M. S., Souto Barcelos M. C., Gomes da Costa J. M. (2018). Microencapsulation and Release Controlled
by the Diffusion of Food Ingredients Produced by Spray Drying: A Review. Braz. J. Food Technol..

[6] Suave J., Dall’agnol E., Pezzin A. P. T., Silva D. A. K., Meier M. M., Soldi V. (2006). Microencapsulação:
Inovação Em Diferentes
Áreas. Revista Saúde e Ambiente.

[7] Krasaekoopt W., Bhandari B., Deeth H. (2003). Evaluation
of Encapsulation Techniques
of Probiotics for Yoghurt. Int. Dairy J..

[8] Falcone G., Saviano M., Aquino R. P., Del Gaudio P., Russo P. (2021). Coaxial Semi-Solid Extrusion and Ionotropic Alginate Gelation: A
Successful Duo for Personalized Floating Formulations via 3D Printing. Carbohydr. Polym..

[9] Azeredo, H. Encapsulação: Aplicação à Tecnologia de Alimentos Alimentos e Nutrição 2008; Vol. 16.

[10] Paula H. C. B. d., Oliveira E. F., Abreu F. O. M. S., Paula R. C. M. d., Morais S. M. d., Forte M. M. C. (2010). Esferas (Beads) de Alginato Como
Agente Encapsulante de Óleo de Croton Zehntneri Pax et Hoffm. Polímeros.

[11] Biedrzycka A., Skwarek E., Hanna U. M. (2021). Hydroxyapatite
with Magnetic Core:
Synthesis Methods, Properties, Adsorption and Medical Applications. Adv. Colloid Interface Sci..

[12] Bonan R. F., Bonan P. R. F., Batista A. U. D., Oliveira J. E., Menezes R. R., Medeiros E. S. (2014). Métodos de
Reforço Microestrutural Da
Hidroxiapatita. Cerâmica.

[13] Modolon H. B., Inocente J., Bernardin A. M., Klegues Montedo O. R., Arcaro S. (2021). Nanostructured Biological Hydroxyapatite from Tilapia
Bone: A Pathway to Control Crystallite Size and Crystallinity. Ceram. Int..

[14] Moretto C. E., Niero A. L. S., Modolon H. B., Teixeira L. B., Demétrio K. B., Arcaro S. (2023). Calcium Phosphate Coating
in Stainless Steel AISI 316L
Using Eletrodeposition for Biological Applications. Material-ES.

[15] Rodovalho A. J. R. L., Barbosa W. T., Vieira J. L., Oliva C. A. d., Gonçalves A. P. B., Cardoso P. d. S. M., Modolon H. B., Montedo O. R. K., Arcaro S., Hodel K. V. S., Soares M. B. P., Ajayan P. M., Barbosa J. D. V. (2024). Influence of Size and Crystallinity of Nanohydroxyapatite
(NHA) Particles on the Properties of Polylactic Acid/NHA Nanocomposite
Scaffolds Produced by 3D Printing. J. Mater.
Res. Technol..

[16] Predoi D., Groza A., Iconaru S. L., Predoi G., Barbuceanu F., Guegan R., Motelica-Heino M. S., Cimpeanu C. (2018). Properties of Basil
and Lavender Essential Oils Adsorbed on the Surface of Hydroxyapatite. Materials.

[17] Militello M., Settanni L., Aleo A., Mammina C., Moschetti G., Giammanco G. M., Blàzquez M. A., Carrubba A. (2011). Chemical Composition
and Antibacterial Potential of Artemisia Arborescens L. Essential
Oil. Curr. Microbiol..

[18] Romero, A. L. ; Oliveira, R. R. ; Romero, R. B. ; Almeida, A. L. ; Diniz, S. P. S. d. S. ; Vida, J. B. Efeito de monoterpenos naturais no crescimento micelial e germinação de conídios de Corynespora cassiicola; Pesquisa Agropecuária Pernambucana 10.12661/pap.2013.002.

[19] Kaur H., Bhardwaj U., Kaur R. (2021). Cymbopogon Nardus Essential
Oil:
A Comprehensive Review on Its Chemistry and Bioactivity. J. Essent. Oil Res..

[20] Nakahara K., Alzoreky N. S., Yoshihashi T., Nguyen H. T. T., Trakoontivakorn G. (2013). Chemical Composition
and Antifungal Activity of Essential Oil from Cymbopogon Nardus (Citronella
Grass). Jpn. Agric. Res. Q..

[21] Wang J., Cai N., Chan V., Zeng H., Shi H., Xue Y., Yu F. (2021). Antimicrobial
Hydroxyapatite Reinforced-Polyelectrolyte Complex Nanofibers
with Long-Term Controlled Release Activity for Potential Wound Dressing
Application. Colloids Surf., A.

[22] Xu Q., Czernuszka J. T. (2008). Controlled
Release of Amoxicillin from Hydroxyapatite-Coated
Poly­(Lactic-Co-Glycolic Acid) Microspheres. J. Controlled Release.

[23] Burduşel A.-C., Sarchizian D., Niculescu A. G., Holban A. M., Popescu R. C., Truşcă R., Andronescu E. (2024). Synthesis and Characterization of
Hydroxyapatite-Zinc Oxide Nanocomposites Incorporating Rosemary and
Thyme Essential Oils for Enhanced Bone Regeneration and Antimicrobial
Activity. Rom. J. Morphol. Embryol..

[24] de
Farias A. L., Meneguin A. B., da Silva Barud H., Brighenti F. L. (2020). The Role of Sodium Alginate and Gellan Gum in the Design
of New Drug Delivery Systems Intended for Antibiofilm Activity of
Morin. Int. J. Biol. Macromol..

[25] Roque-Borda C. A., Pereira L. P., Guastalli E. A. L., Soares N. M., Mac-Lean P. A. B., Salgado D. D., Meneguin A. B., Chorilli M., Vicente E. F. (2021). HPMCP-Coated
Microcapsules Containing the Ctx­(Ile21)-Ha Antimicrobial Peptide Reduce
the Mortality Rate Caused by Resistant Salmonella Enteritidis in Laying
Hens. Antibiotics.

[26] Brazil . Lei No 11.794, de 8 de Outubro de. Regulamenta a Criação e a Utilização de Animais Em Atividades de Ensino e Pesquisa Científica Brasília; 2008 https://www.planalto.gov.br/ccivil_03/_ato2007-2010/2008/lei/l11794.htm (accessed Nov 23, 2025).

[27] Ministério da Ciência, Tecnologia e Inovação, CONCEA . Guia Brasileiro de Produção, Manutenção Ou Utilização de Animais Em Atividades de Ensino Ou Pesquisa Científica Brasília, Brasil; 2023 https://www.gov.br/mcti/pt-br/composicao/conselhos/concea/arquivos/arquivo/publicacoes-do-concea/guia_concea_1ed_animais-_ensino_ou_pesquisa_2023.pdf (accessed Nov 23, 2025).

[28] Lucia
de Souza Niero A., Possolli N. M., Floriano da Silva D., Demétrio K. B., Zocche J. J., Soares de Souza G. M., Dias J. F., Vieira J. L., Viana Barbosa J. D., Pereira Soares M. B., Klegues Montedo O. R., Arcaro S. (2023). Composite Beads of
Alginate and Biological Hydroxyapatite from Poultry and Mariculture
for Hard Tissue Repair. Ceram. Int..

[29] Bauer A. W., Kirby W. M. M., Sherris J. C., Turck M. (1966). Antibiotic Susceptibility
Testing by a Standardized Single Disk Method. Am. J. Clin. Pathol..

[30] Bezerra F. M., Carmona O. G., Carmona C. G., Lis M. J., de Moraes F. F. (2016). Controlled
Release of Microencapsulated Citronella Essential Oil on Cotton and
Polyester Matrices. Cellulose.

[31] Pratiwi L., Eddy D. R., Al Anshori J., Harja A., Wahyudi T., Mulyawan A. S., Julaeha E. (2022). Microencapsulation of Citrus Aurantifolia
Essential Oil with the Optimized CaCl2 Crosslinker and Its Antibacterial
Study for Cosmetic Textiles. RSC Adv..

[32] Lu X., Guo W., Wang B., Feng Y., He S., Xue L. (2023). Screening
Optimal Preparation Conditions of Low-Cost Metal-Modified Biochar
for Phosphate Adsorption and Unraveling Their Influence on Adsorption
Performance. J. Cleaner Prod..

[33] Zheng C., Yang X., Li M., Bai S. (2024). Bridging the Adsorption
Data and Adsorption Process by Introducing a Polynomial Structure
To Accurately Describe IUPAC Isotherms, Stepwise Isotherms, and Stepwise
Breakthrough Curves. Langmuir.

[34] Chen R., Shi J., Zhu B., Zhang L., Cao S. (2020). Mesoporous Hollow Hydroxyapatite
Capped with Smart Polymer for Multi-Stimuli Remotely Controlled Drug
Delivery. Microporous Mesoporous Mater..

[35] Li D., Huang X., Wu Y., Li J., Cheng W., He J., Tian H., Huang Y. (2016). Preparation
of PH-Responsive Mesoporous
Hydroxyapatite Nanoparticles for Intracellular Controlled Release
of an Anticancer Drug. Biomater Sci..

[36] Lin Y. W., Fang C. H., Liang Y. J., Yang C. Y., Kuo W. T., Lin F. H. (2023). Controlled Release
of Clenbuterol from a Hydroxyapatite
Carrier for the Treatment of Alzheimer’s Disease. Biomater Res..

[37] Mendes
da Silva C., Lins Silva T., Macário
Pinto I. (2019). Caracterização Reológica
de Fluidos Não Newtonianos e Sua Aplicabilidade Na Indústria. Ciências exatas tecnol..

[38] Jafari S. M., Assadpoor E., He Y., Bhandari B. (2008). Encapsulation Efficiency
of Food Flavours and Oils during Spray Drying. Drying Technol..

[39] Corrêa N. M., Bueno F., Júnior C., Fernanda Ignácio R., Leonardi G. R. (2005). Avaliação
Do Comportamento Reológico
de Diferentes Géis Hidrofílicos. Rev. Bras. Cienc. Farm..

[40] Reineccius G. A., Bangs W. E. (1985). Spray Drying of
Food Flavors. Ill. Optimum Infeed Concentrations
for the Retention of Artificial Flavors. Perfum.
Flavor..

[41] Acharya S., Jakeer S., Shilpa P., Andhale S. (2021). Review: Flavor Encapsulation
by Spray Drying Technique. Int. J. Chem. Stud..

[42] Niero, A. L. D. S. ; Arcaro, S. Esferas de Alginato/Nanohidroxiapatita Obtida a Partir de Subprodutos Da Avicultura e Da Maricultura Para Reparo Tecidual; Trabalho de Conclusão de Curso, Universidade do Extremo Sul Catarinense: Criciúma, 2022.

[43] Lopes W. A., Fascio M. (2004). Esquema Para Interpretação De Espectros
De Substâncias Orgânicas Na Região Do Infravermelho. Quim. Nova.

[44] Silverstein, M. ; Webster, F. ; Kiemle, D. J. Spectrometric Identification of Organic Compounds, 7th ed.; John Wiley & Sons, Inc: New York, 2005.

[45] Brugnera D. F., de Oliveira M. M. M., Piccoli R. H. (2011). Essential Oils of
Cymbopogon Sp.
in the Control of Foodborne Pathogenic Bacteria. Alimentos e Nutrição.

[46] El
Kamari F., Taroq A., Atki Y. El., Aouam I., Lyoussi B., Abdellaoui A. (2018). Chemical Composition of Essential
Oils from Vitex Agnus-Castus l. Growing in Morocco and Its in Vitro
Antibacterial Activity against Clinical Bacteria Responsible for Nosocomial
Infections. Asian J. Pharm. Clin. Res..

[47] EL
Kamari F., Taroq A., El Atki Y., Aouam I., Oumokhtar B., Lyoussi B., Abdellaoui A. (2018). Cymbopogon
Nardus L. Essential Oil: Phytochemical Screening and Its Antibacterial
Activity against Clinical Bacteria Responsible for Nosocomial Infections
in Neonatal Intensive Care. Int. J. Pharm. Sci.
Rev. Res..

[48] Wei L. S., Wee W. (2013). Chemical Composition
and Antimicrobial Activity of Cymbopogon Nardus
Citronella Essential Oil against Systemic Bacteria of Aquatic Animals. Iran J. Microbiol..

[49] Sari I., Misrahanum M., Faradilla M., Faradilla M., Mutia Ayuningsih C., Ayuningsih C. M., Maysarah H. (2022). Antibacterial Activity
of Citronella Essential Oil from Cymbopogon Nardus (L.) Rendle) Against
Methicillin- Resistant Staphylococcus Aureus. Indones. J. Pharm. Clin. Res..

[50] Aparecida
Andrade M., das Graças Cardoso M., Roberto
Batista L., Cristina Teixeira Mallet A., Maria Fernandes
Machado S. (2012). Essential Oils of Cinnamomum Zeylanicum, Cymbopogon
Nardus and Zingiber Officinale: Composition, Antioxidant and Antibacterial
Activities. Rev. Cienc. Agron..

[51] Bizerra A., Silva V. (2016). Sistemas De Liberação
Controlada: Mecanismos e Aplicações. Rev. Saúde Meio Ambiente.

[52] Wolfe, M. S. ; Scopazzi, C. Rheology of Swellable Microgel Dispersions: Influence of Crosslink Density 1. 1989.

[53] Solomon B., Sahle F. F., Gebre-Mariam T., Asres K., Neubert R. H. H. (2012). Microencapsulation
of Citronella Oil for Mosquito-Repellent Application: Formulation
and in Vitro Permeation Studies. Eur. J. Pharm.
Biopharm..

[54] Aguiar M. C. S., das Graças Fernandes da Silva M.
F., Fernandes J. B., Forim M. R. (2020). Evaluation of the Microencapsulation of Orange Essential
Oil in Biopolymers by Using a Spray-Drying Process. Sci. Rep..

[55] Abreu F. O. M. S., Oliveira E. F., Paula H. C. B., De Paula R. C. M. (2012). Chitosan/Cashew
Gum Nanogels for Essential Oil Encapsulation. Carbohydr. Polym..

[56] Zhdanov V. P. (2023). Release
of Molecules from Nanocarriers. Phys. Chem.
Chem. Phys..

[57] Moshe I., Weizman O., Natan M., Jacobi G., Banin E., Dotan A., Ophir A. (2016). Multiphase Thermoplastic Hybrid for
Controlled Release of Antimicrobial Essential Oils in Active Packaging
Film. Polym. Adv. Technol..

[58] Zhang T., Luo Y., Wang M., Chen F., Liu J., Meng K., Zhao H. (2020). Double-Layered
Microcapsules Significantly Improve the Long-Term
Effectiveness of Essential Oil. Polymers.

