# Supplementary material for: Tilapia Bone-Derived Hydroxyapatite Particles for Controlled Citronella (Cymbopogon nardus) Release and Antimicrobial Activity
Source: ACS Omega. 2026 Feb 12;11(7):12009–21. doi: 10.1021/acsomega.5c11133 (PMC12947014; doi:10.1021/acsomega.5c11133)
Supplement: Supplementary file 1 [file ao5c11133_si_001.pdf]

# Tilapia Bone-Derived Hydroxyapatite Particles for Controlled Citronella (*Cymbopogon nardus*) Release and Antimicrobial Activity

Janaina Tasca Serafim[a*], Henrique Borba Modolon[a], Natália Morelli Possolli[a], Oscar R. K. Montedo[a], Elídio Angioletto[b], Maria Alice Prado Cechinel[c], Sabrina Arcaro[a]

[a] *Technical Ceramics Laboratory (CerTec), Biomaterials and Nanostructured Materials Group, Graduate Program in Materials Science and Engineering (PPGCEM), University of the Extreme South of Santa Catarina, 88806-000, Criciúma (SC), Brazil*

[b] *Laboratory of Biomaterials and Antimicrobial Materials Development (LADEBIMA), University of the Extreme South of Santa Catarina, 88806-000, Criciúma (SC), Brazil*

[c] *Department of Chemical and Food Engineering (EQA), Federal University of Santa Catarina (UFSC), 88040-900, Florianópolis (SC), Brazil*

***Corresponding author:**

Janaina Tasca Serafim, janaina.tasca@unesc.net, ORCID: 0000-0002-2412-5516

## Supplementary Material

**Figure S1.** Cross-sectional micrographs of particles with 500× magnification: (a) Sample Alg-EO-H0.075, (b) Sample Alg-EO-H0.15

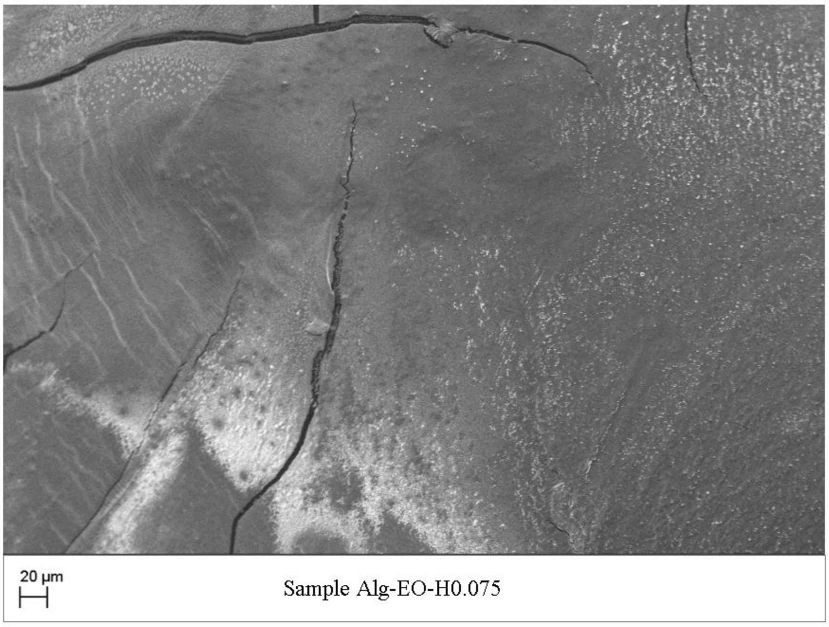


(a)

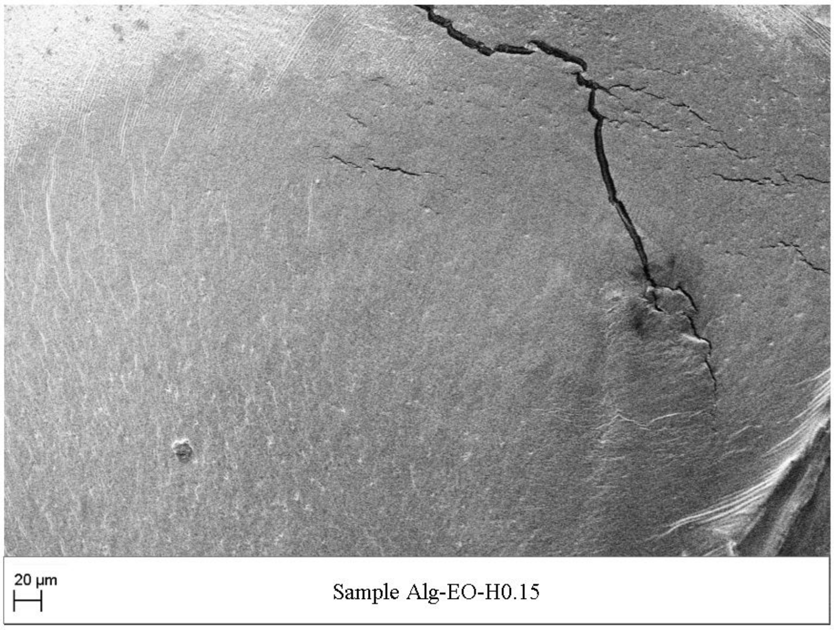


(b)

**Table S1.** Two-way ANOVA results for physical properties (bulk density, relative density, and porosity)

| Evaluated variable | Main factors / Interaction | p-value |
|---|---|---|
| Physical properties | True density, relative density, and porosity | < 0.0001 |
| | HA concentration | 0.0955 |

**Table S2.** Two-way ANOVA results for particle diameter at different exposure times and HA concentrations

| Evaluated variable | Main factors / Interaction | p-value |
|---|---|---|
| Particle diameter | HA concentration | < 0.0001 |
| | Exposure time | < 0.0001 |
| | HA × time interaction | < 0.0001 |

**Table S3.** Two-way ANOVA results for essential oil release as a function of time and HA concentration.

| Evaluated variable | Main factors / Interaction | p-value |
|---|---|---|
| Release of essential oil | Release time | 0.0425 |
| | HA concentration | 0.0001 |
| | Time × concentration interaction | <0.0001 |
